# Strategic prioritisation enhances young and older adults’ visual feature binding in working memory

**DOI:** 10.1177/1747021820960712

**Published:** 2020-09-30

**Authors:** Richard J Allen, Amy L Atkinson, Louise A Brown Nicholls

**Affiliations:** 1 School of Psychology, University of Leeds, Leeds, UK; 2 Centre for Applied Education Research, Wolfson Centre for Applied Health Research, Bradford Royal Infirmary, Bradford, UK; 3 School of Psychological Sciences & Health, University of Strathclyde, Glasgow, UK

**Keywords:** Visual working memory, cognitive ageing, strategy, prioritisation, processing speed

## Abstract

Visual working memory for features and bindings is susceptible to age-related decline. Two experiments were used to examine whether older adults are able to strategically prioritise more valuable information in working memory and whether this could reduce age-related impairments. Younger (18–33 years) and older (60–90 years) adults were presented with coloured shapes and, following a brief delay, asked to recall the feature that had accompanied the probe item. In Experiment 1, participants were either asked to prioritise a more valuable object in the array (serial position 1, 2, or 3) or to treat them all equally. Older adults exhibited worse overall memory performance but were as able as younger adults to prioritise objects. In both groups, this ability was particularly apparent at the middle serial position. Experiment 2 then explored whether younger and older adults’ prioritisation is affected by presentation time. Replicating Experiment 1, older adults were able to prioritise the more valuable object in working memory, showing equivalent benefits and costs as younger adults. However, processing speed, as indexed by presentation time, was shown not to limit strategic prioritisation in either age group. Taken together, these findings demonstrate that, although older adults have poorer visual working memory overall, the ability to strategically direct attention to more valuable items in working memory is preserved across ageing.

Adult ageing is typically associated with relatively stable or increased crystallised intelligence (e.g., verbal knowledge, wisdom; Ardelt, 2010; D. C. Park et al., 2002) along with declining fluid cognitive abilities, including processing speed, executive functioning, and short-term (“working”) memory (Hedden & Gabrieli, 2004; Reuter-Lorenz & Lustig, 2016). Visual working memory, the ability to temporarily process and store visual material, appears to be particularly sensitive to ageing (Johnson et al., 2010; Logie & Maylor, 2009; Murre et al., 2013; Swanson, 2017). This has been found for a variety of visual stimuli such as black and white matrix patterns, and basic colours, shapes, or orientations (e.g., Beigneux et al., 2007; Brockmole & Logie, 2013; Brown et al., 2017; Hamilton et al., 2018; Logie & Maylor, 2009; Nicholls & English, 2020; Peich et al., 2013). Furthermore, older adults have a reduced ability to retain both individual visual features in working memory (such as colours or shapes) and their associations (“bindings”; Allen et al., 2013; Brockmole et al., 2008; Brown & Brockmole, 2010; Brown et al., 2017; Chen & Naveh-Benjamin, 2012; Guazzo et al., 2020; Peich et al., 2013; Peterson & Naveh-Benjamin, 2016, 2017; Read et al., 2016; Rhodes et al., 2016, 2017).

There are several suggested mechanisms for these age-related declines. Neural representations, including in the visual cortex, have been shown to be less specific or distinct with ageing (e.g., D. C. Park et al., 2004; J. Park et al., 2010; Payer et al., 2006; Spreng et al., 2010). Similarly, older adults may store and/or recall information with less resolution or precision, which has been shown to be more problematic with larger arrays (Ko et al., 2014; Noack et al., 2012; Peich et al., 2013; Pertzov et al., 2015). Another hypothesis is that older adults have poorer working memory due to reduced executive attentional capacity (Braver & West, 2008; Reuter-Lorenz & Lustig, 2016), with visuo-spatial executive functioning accounting for significant variance in older adults’ visual working memory capacity (Brown et al., 2012). Age-related under-recruitment of frontal cortex has also been observed during intentional memory encoding (Logan et al., 2002); however, “age-related dedifferentiation” may reflect compensatory processes and age-related neural reorganisation (Bock et al., 2019; Koen & Rugg, 2019). A further possibility is that visual working memory undergoes lifespan changes in separate systems responsible for the formation of representations, and their active maintenance (Ozimič & Repovš, 2020). Under this approach, healthy ageing is associated with declines both in the ability to establish a distinct number of visual representations and in actively maintaining all or some of these representations following their offset from the environment.

Recent evidence suggests that young adults are able to direct attentional resources to prioritise certain items in visual working memory, but little is known about strategic use of attention in older people, and its potential to enhance visual working memory. Given the hypothesis above, that older adults experience an executive deficit, it is possible that ageing may reduce the ability to prioritise information in working memory. Here, we address prioritisation in the context of age-sensitive visual working memory, with the aim of establishing its impacts on healthy older adults’ capacity. Visual binding was focused on, as this is typically more challenging than individual feature memory, especially in the context of sequentially presented arrays, both for young and older people (e.g., Brown et al., 2017).

## Strategic prioritisation

Young adults’ ability to use value-based prioritisation to enhance visual binding recall is relatively well understood. Young adults can prioritise more valuable information in working memory, which subsequently enhances memory for that item if it is tested (Allen & Ueno, 2018; Atkinson, Berry, et al., 2018; Hitch et al., 2018; Hu et al., 2014, 2016; Sandry et al., 2014). This enhancement can be observed when applied to different positions in a four-item sequence. It typically emerges in the form of a serial position × prioritisation interaction, rather than a main effect of prioritisation overall, with boosts to the high-value items offset by performance decrements to other, lower value items in the sequence. This therefore indicates how participants are able to strategically redistribute limited attentional resources to different items in a sequence. It is also normally observed alongside a recency advantage to the final item, regardless of which item is being prioritised, reflecting the contribution of both internally directed, controlled attention and externally captured, automatic attentional influences. Prioritised items are assumed to be held in a privileged, accessible state in working memory, possibly synonymous with a focus of attention and/or episodic buffer. The benefits that are observed for such items are however vulnerable to different forms of attentional interference, both in the form of executive-attentional load and perceptual interference from the environment (see Hitch et al., 2020, for a review).

Developmental research has shown that children can direct their attention to more valuable information in working memory although, relative to young adults, they appear to need extra motivation to do so (Atkinson et al., 2019). When such motivation is absent, there is evidence of no prioritisation effect (Berry et al., 2018). Taken together, this suggests that children are able to prioritise more valuable information in working memory, but only in some task contexts. Research to date has not, however, investigated whether the ability to implement value-directed attentional prioritisation in working memory is retained into older adulthood. Given that working memory is reduced in older adults, the ability to selectively prioritise more valuable information is likely to be particularly beneficial to this group. However, given age-related limitations in executive functioning, it is questionable whether older adults can prioritise as well as younger people. The current experiments examined this.

There is some evidence that older people can direct their attention to particular items (Atkinson, Baddeley, et al., 2018; Gilchrist et al., 2016; Loaiza & Souza, 2018; Mok et al., 2016; Strunk et al., 2019; Souza, 2016) or tasks (Rhodes et al., 2019) within working memory. For instance, Atkinson, Baddeley, et al. (2018) found that focusing on some items resulted in better performance relative to a condition in which participants tried to remember all of the items when four or six items were presented. This effect did not differ as a function of age. Furthermore, some visual cueing studies which direct participants towards one or more particular item(s) have reported that older adults experience similar sized cueing effects to younger adults (Gilchrist et al., 2016; Loaiza & Souza, 2018; Mok et al., 2016; Souza, 2016; Strunk et al., 2019). For example, Souza (2016) found that pre-cues and retro-cues enhanced performance to similar magnitudes in younger and older adults, relative to a condition in which no cue was presented. Similarly, Strunk et al. (2019) found that, although older adults exhibited worse overall performance, retro-cues enhanced working memory and long-term memory to a similar extent in young adults and older adults. Based on such findings, it might be predicted that individuals would be able to prioritise more valuable information in visual working memory as effectively as younger adults.

Several studies have, however, found that older adults are somewhat impaired in their ability to direct attention in memory relative to younger adults (Castel et al., 2011; Duarte et al., 2013; Newsome et al., 2015; Yi & Friedman, 2014). For instance, within long-term memory, it has been found that older adults are able to prioritise more valuable information (Castel et al., 2009, 2011; Siegel & Castel, 2018), although this ability is reduced in old-old adults (*M* = 85 years) relative to younger adults (Castel et al., 2011). Some studies have also found that older adults are less able to direct their attention in working memory relative to young adults. Duarte et al. (2013) found that young adults, but not older adults, benefitted from retro-cues in a visual working memory task. Similarly, Newsome et al. (2015) found that young adults, patients with mild cognitive impairment, and patients with medial temporal lobe amnesia benefitted from retro-cues, while healthy older adults did not. Finally, in an examination of selective attention using a visual search task, Störmer et al. (2014) found that older adults did show a search advantage for high reward items, though this was somewhat less pronounced and consistent than that observed in young adults.

Given these inconsistent findings, and the generally limited research on the role of strategy in older adults’ working memory (Lemaire, 2016), further research is needed to establish whether healthy older adults can successfully direct their attention in working memory. This will help inform debates on cognitive ageing and the relationship between working memory and attention. It also has practical implications regarding possible provision of guidance and support in how to optimise the goal-directed efficiency of a limited capacity working memory system that declines with age. Two experiments were therefore carried out. If older adults have limited executive resources, they may be less able to direct their attention in working memory relative to young adults and exhibit less of a performance boost from strategic prioritisation as a result. Experiment 1 therefore examined whether young and older adults show equivalent or differential ability to prioritise items, based on their value, from different positions in a three-item sequence. Experiment 2 then sought to replicate the observed patterns at serial position 2 and additionally examined whether the magnitude of prioritisation benefits produced by each age group are influenced by variation in encoding time.

## Experiment 1

Experiment 1 was designed to assess young and older adults’ ability to prioritise items in visual working memory. While young adults have consistently shown this ability, there is currently very limited understanding of how effectively older adults are able to prioritise a particular item from a to-be-remembered visual sequence. One possibility is that older adults have a reduced ability to do so, due to age-related decline in executive attentional resources assumed to be important in underlying value-based prioritisation (Hu et al., 2016). Indeed, without strategy instruction, older adults may not be as flexible as young adults in deploying various strategies in visual working memory (Hamilton et al., 2018; Nicholls & English, 2020). However, with strategic instruction to focus attention towards certain items, older adults may benefit just as much as young adults from prioritising the high-value item in a visual sequence. In the visual working memory domain, Atkinson, Baddeley, et al. (2018) showed that both young and older adults benefitted from the instruction to focus on a subset of items in the array. Also, both age groups have been shown to benefit from value-based importance in visuo-spatial and verbal associative long-term memory (Ariel et al., 2015; Hennessee et al., 2018; Siegel & Castel, 2018). It is not yet known if this same benefit would be observed in visual working memory, when participants are asked to focus on one particular, high-value item. However, based on beneficial value-directed attentional selection in associative memory, it could be predicted that both young and older adults can implement and benefit from a value-based strategy in visual working memory.

This was examined across each of the three serial positions in the sequence. Previous work with young adults has demonstrated value-based prioritisation improvements in accuracy for any serial position in a visual sequence (Hitch et al., 2018; Hu et al., 2014, 2016). However, it is also the case that the involvement of controlled versus automatic attentional components may vary as a function of where in a sequence an item appeared. Earlier sequence items are typically more vulnerable to loss and more dependent on executive attention, relative to the final sequence item (Allen et al., 2006, 2014) and are potentially more likely to exhibit a benefit from prioritisation. Particularly for three-item sequences as used in the present research, the middle item has been observed to be particularly vulnerable, especially for older adults. In a recognition (change detection) paradigm involving 3-object sequential arrays, Brown et al. (2017; Exp. 2) showed that, while young adults exhibited no effect of serial position, older adults’ performance was relatively poor (almost at chance level) at position 2 (for the same pattern in verbal working memory/mental arithmetic tasks, see Foos, 1989; Foos & Wright, 1992). Experiment 1 therefore explored whether any age-related changes in the magnitude of prioritisation effects might vary with sequence position.

### Method

#### Participants

Prior to commencing data collection, the experiment was approved by the Ethics Committee of the School of Psychological Sciences & Health at the University of Strathclyde (approval number 516). Power analysis (G*Power 3; Faul et al., 2007) was carried out, with a focus on the difference between equal value and high value trials at the targeted serial positions. Based on observing a large effect (*d* = .80; see Atkinson, Berry, et al., 2018, Exp. 1) with 80% power at α = .05, this indicated a required sample size of 15 participants per group. There were 48 participants in total, equivalent to similar published studies (e.g., Brown et al., 2017; Rhodes et al., 2016). The young age group included 14 males and 10 females, primarily recruited from the University of Strathclyde student population, who received course credit for participation. They were aged 19–33 years (*M* = 23.5, *SD* = 3.87) and their mean number of years in full-time education was 14.54 (*SD* = 1.67). Their mean estimated full-scale IQ was 106.63 (*SD* = 5.07; based on the National Adult Reading Test, Nelson & Willison, 1991). The older adults volunteered to participate on the basis of being healthy and living independently and received no incentives. They all passed screening for dementia using the Mini-Cog (Borson et al., 2000). They included 7 males and 17 females, aged 60–87 years (*M* = 71.54, *SD* = 6.91) with a mean number of years of education of 16.04 (*SD* = 1.46), and a mean estimated IQ of 113.25 (*SD* = 3.76). Years of education, *t*(46) = 3.32, *p* = .002, and estimated IQ, *t*(46) = 5.14, *p* < .001, were significantly higher in the older adults, and in the opposite direction of any expected age effects on memory. All participants reported vision correction if required, and no memory impairments.

#### Design

The design took the form of a 2 (age group—young, older) × 4 (prioritisation—control, or prioritise serial positions 1, 2, or 3; repeated measures) × 3 (serial position—1, 2, 3; repeated measures) mixed factorial design. Performance was measured by the proportion of trials correct.

#### Materials

##### Visual binding task

**Figure 1.**
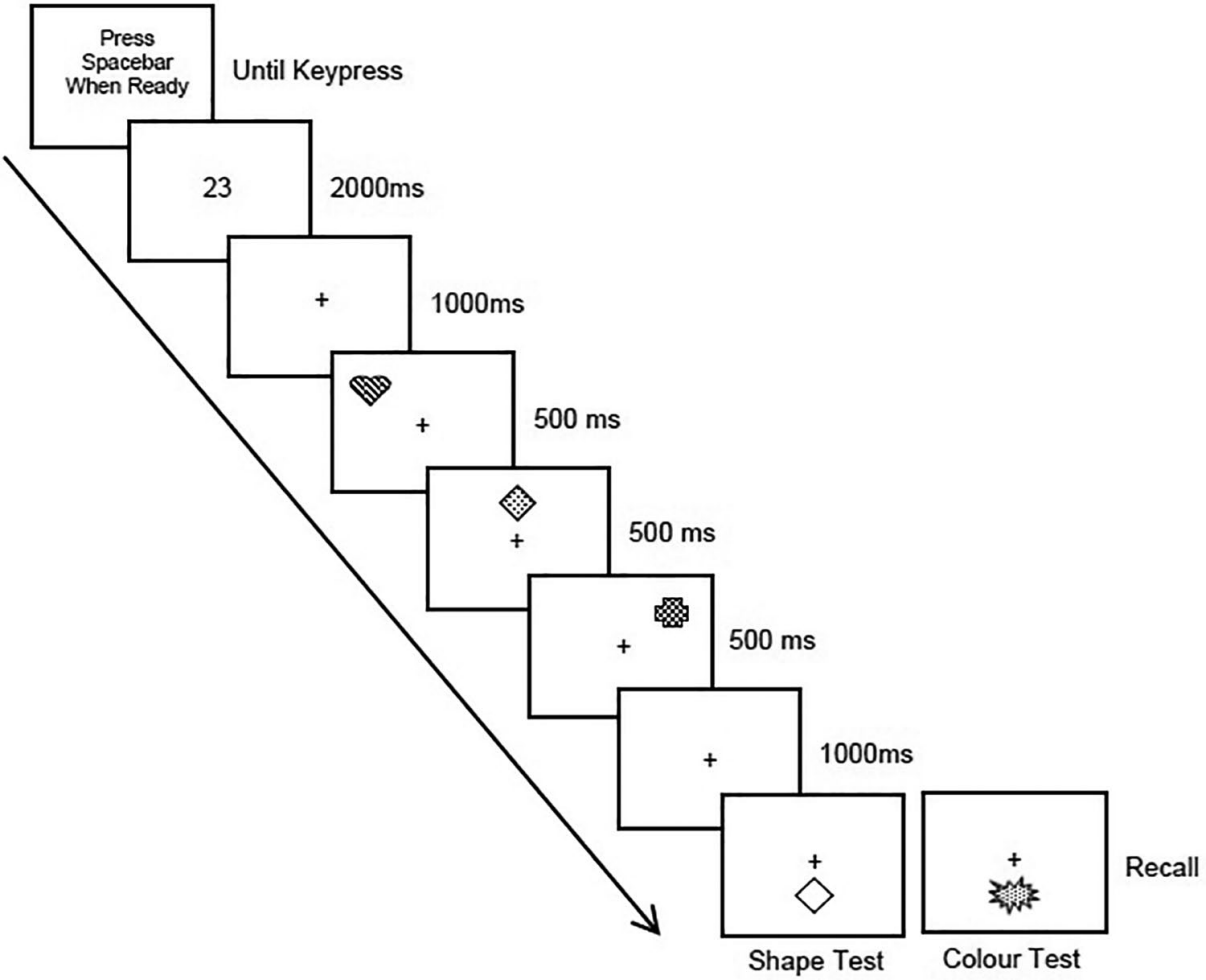
An example trial from the paradigm used in Experiment 1. Participants were sequentially presented with three coloured shapes to remember, and memory was tested either by presenting a shape or colour probe. Participants were asked to recall out loud the accompanying feature. Articulatory suppression was carried out from the beginning of each trial until the verbal response was made at the end. Note, different fill effects depict different colours, and stimuli are not drawn to scale.

A visual feature binding task was administered to participants via E-Prime 2.0 (Psychology Software Tools, Inc.; see Figure 1) and this involved cued verbal recall (e.g., Hu et al., 2014). Memory stimulus arrays comprised three coloured shapes presented on a grey background. Each object was created from a pool of six colours (red, yellow, blue, green, cyan, purple) and six shapes (circle, triangle, diamond, heart, arrow, cross), by randomly selecting one colour and one shape, without replacement. Each stimulus measured approximately 2 cm^2^ on screen, and viewing distance was not constrained. There were four blocks of trials, paired equally often with each prioritisation condition. In each block there were 30 trials in total, 10 testing each serial position. Half of the test probes testing each position in each block comprised a black outline of a shape, with participants asked to recall the accompanying colour. The other half comprised colour “blobs,” probing the accompanying shape. Each trial block began with six practice trials. Within each set of practices, half of the probes were colours while the other half were shapes, and each serial position was probed twice. Trial feedback was never provided, either within the practices or the experimental trials.

##### Prioritisation instructions

For each prioritisation condition, participants were either asked to try equally hard to remember each object (control condition) or to try extra hard to remember a particular object (i.e., first, middle, or last). In all conditions, participants were informed that they may be asked to recall the first, second, or third object in any given trial, regardless of the prioritisation instruction, and that each object would be tested an equal number of times. In the control condition, participants were informed that they would gain 1 point if they were asked about any item and they responded correctly. In the prioritisation conditions, participants were advised that they would get more points (4) if they were asked about the prioritised object and they got the answer right, and 1 for the other serial positions in the sequence.

##### Cognitive screening and IQ estimation

The National Adult Reading Test (NART) was used to estimate IQ (Nelson & Willison, 1991), which involved participants reading out loud a list of 50 words that were progressively less frequent and more difficult to pronounce. Participants were asked to attempt to pronounce all items and to guess if they were unsure, as this was part of the task. The Mini-Cog (Borson et al., 2000) was used to screen the older adults for signs of unhealthy cognitive decline. This involved assessing verbal recall and clock drawing ability.

#### Procedure

All participants gave written, informed consent prior to participation. Older participants first completed the Mini-Cog (Borson et al., 2000). All participants completed the NART (Nelson & Willison, 1991) before then carrying out the computerised memory task. Trials were administered in four blocks paired with the four prioritisation conditions, the order of which was counterbalanced (see Figure 1). Participants began a given trial by pressing the space bar, after which a 2-digit number was presented on screen for 2,000 ms. Participants were asked to articulate this number out loud, consistently and at the pace of approximately two repetitions per second, until they were ready to give their verbal response at the end of the trial.^1^ The purpose of this was to suppress articulation of the visual stimuli (Baddeley, 2007). After the number disappeared there was a 1,000 ms delay before the memory array was presented. Each object was presented one after the other, for 500 ms each, across the top of the screen from left to right. There was then a 1,000 ms delay before the test probe was presented (blank shape or blob of colour), and participants were asked to report the feature that had accompanied the test item within the array. A cross was presented on the centre of the screen throughout the trial, and participants were asked to fixate on this for the trial duration. In each trial, the experimenter recorded the number of articulations and the recalled item. During debriefing, participants were advised that their performance was not actually being scored using the points system contained within the strategy instructions (i.e., 1 vs 4 points) and that this was used to help explain and encourage the intended prioritisation strategy within each condition.

### Results

**Figure 2.**
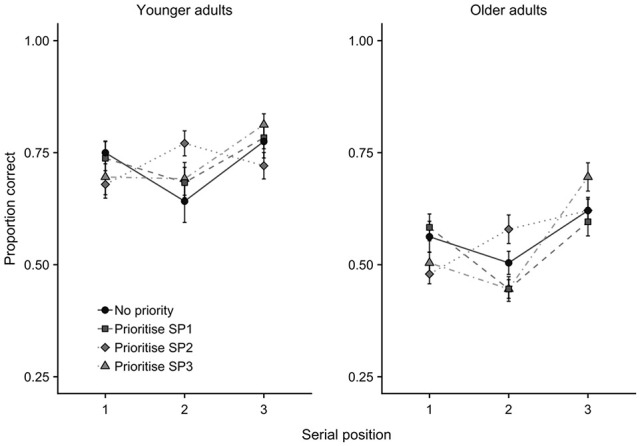
Mean proportion correct data (with *SE*) for each age group in Experiment 1, as a function of priority condition and serial position.

All analysis was carried out using JASP 0.11.1.0 (JASP Team, 2019; Wagenmakers et al., 2018). Data resulting from shape and colour test probes were collapsed together to gain an overall measure of accuracy by age group, prioritisation condition, and serial position (see Figure 2). A 2 × 4 × 3 mixed analysis of variance (ANOVA) was used to analyse the data, with greenhouse-geisser correction used where sphericity could not be assumed, and Bonferroni-Holm correction used in all follow-up multiple comparisons. In addition to frequentist analysis outcomes, Bayes factors are also reported. These provide an estimation of the strength of evidence for the data under the null versus the alternate hypotheses. For ANOVA outcomes, these correspond to BF_incl_, that is, the strength of evidence for the inclusion of each factor and interaction in the model. For follow-up comparisons, BF_10_ are reported, indicating the evidence for the presence of an effect. In each case, BF < 1 indicates support for the null hypothesis, and BF > 1 support for the alternative hypothesis. While Bayes factors should be interpreted as a continuous outcome, we refer to the classification scheme in which BF 1–3 equates to weak or anecdotal evidence, BF 3–10 as moderate evidence, and BF > 10 as strong evidence (Jeffreys, 1961; Lee & Wagenmakers, 2014).

**Figure 3.**
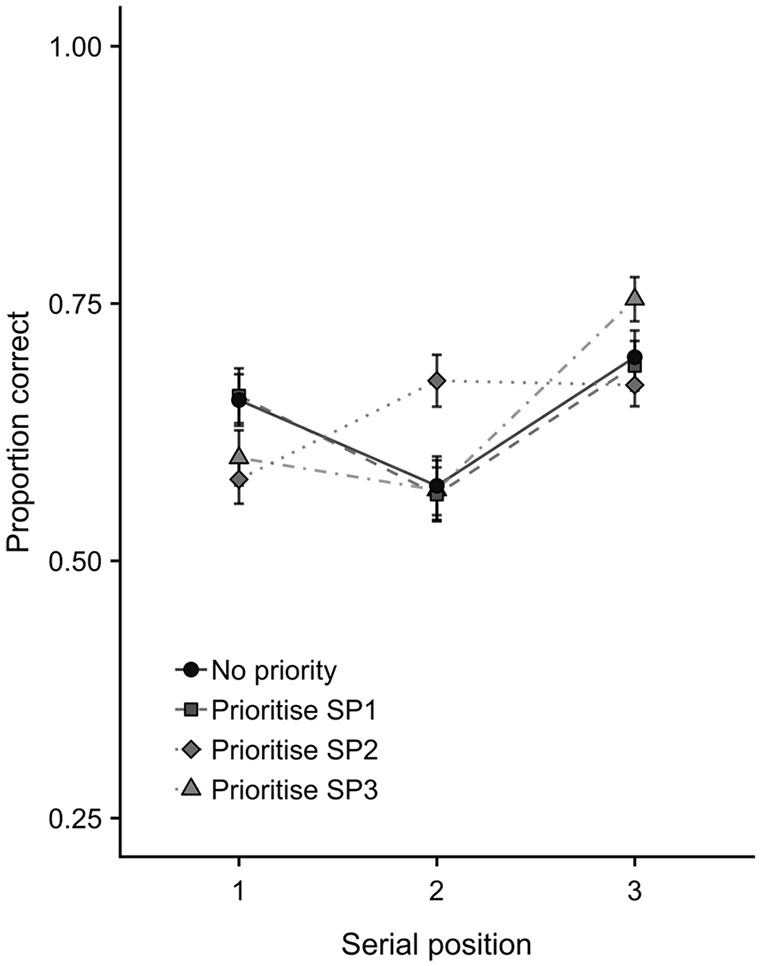
The significant interaction between priority condition and serial position on mean proportion correct (with *SE*) from Experiment 1.

The ANOVA showed main effects of age group, *F*(1,46) = 47.81, *MSE* = 0.093, *p* < .001, $\eta_{p}^{2}=.51$, *BF* > 10,000, in which the young adults (*M* = 0.72, *SE* = 0.02) outperformed the older adults (*M* = 0.55, *SE* = 0.02), and of serial position, *F*(1.6,74.6) = 21.58, *MSE* = 0.034, *p* < .001, $\eta_{p}^{2}=.32$, *BF* > 10,000, in which performance at position 2 (*M* = 0.60, *SE* = 0.02) was poorer than at both positions 1 (*M* = 0.62, *SE* = 0.02), *t*(47) = 2.33, *p* = .024, *d* = .34, *BF* = 1.79, and 3 (*M* = 0.70, *SE* = 0.02), *t*(47) = 5.41, *p* < .001, *d* = .78, *BF* = 8,195. Performance at position 1 was also poorer than at position 3, *t*(47) = 4.34, *p* < .001, *d* = .63, *BF* = 302. There was no overall effect of prioritisation condition, *F*(2.65,121.84) = 0.04, *MSE* = 0.02, *p* = .99, $\eta_{p}^{2}=.00$, *BF* = 0.007. However, there was a significant interaction between prioritisation and serial position, *F*(6,276) = 9.37, *MSE* = 0.015, *p* < .001, $\eta_{p}^{2}=.17$, *BF* > 10,000 (see Figure 3). There were no other two-way, age group × serial position, *F*(1.62,74.57) = 1.83, *MSE* = 0.03, *p* = .17, $\eta_{p}^{2}=.04$, *BF* = 0.555; age group × prioritisation, *F*(2.65,121.84) = 0.70, *MSE* = 0.02, *p* = .54, $\eta_{p}^{2}=$.02, *BF* = 0.032, or 3-way, age group × serial position × prioritisation, *F*(5.10,234.72) = 1.48, *MSE* = 0.02, *p* = .20, $\eta_{p}^{2}=.03$, *BF* = 0.133, interactions.

**Figure 4.**
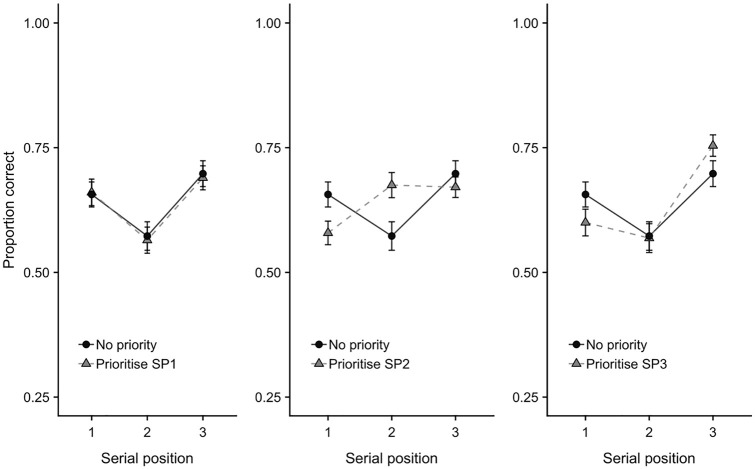
Mean proportion correct (with *SE*) in each priority condition contrasted with the control (no priority) condition from Experiment 1. Data are presented by serial position.

The prioritisation × serial position interaction was followed up with three 2 × 3 ANOVAs comparing the control (no priority) condition with each of the prioritisation conditions in turn (see Figure 4). Comparing no priority with prioritise-SP1 indicated no condition × position interaction, *F*(2,92) = 0.08, *MSE* = 0.02, *p* = .20, $\eta_{p}^{2}=.04$, *BF* = 0.076. In contrast, for prioritise-SP2, the condition × position interaction was significant, *F*(2,92) = 15.55, *MSE* = 0.01, *p* < .001, $\eta_{p}^{2}=.04$, *BF* = 3,887. This was broken down using Bonferroni-Holm corrected *t*-tests comparing no priority versus prioritise-SP2 conditions at each serial position. Performance in the prioritise-SP2 condition was less accurate (i.e., a prioritisation cost relative to no priority trials) at SP1, *t*(47) = 3.48, *p* = .002, *d* = .50, *BF* = 26.89, more accurate (i.e., a prioritisation benefit) at SP2, *t*(47) = 3.95, *p* < .001, *d* = .57, *BF* = 95.85, and did not differ from no priority at SP3, *t*(47) = 1.17, *p* > .05, *d* = .17, *BF* = 0.297. Finally, there was also a condition × serial position interaction for the comparison between no priority and prioritise-SP3, *F*(2,92) = 4.62, *MSE* = 0.02, *p* = .012, $\eta_{p}^{2}=.04$, *BF* = 2.45, though this was not well supported by Bayesian analysis, and Bonferroni-Holm corrected *t*-tests indicated no clear evidence of differences between no priority and prioritise-SP3 at any serial position (SP1, *d* = .28, *BF* = 0.89; SP2, *d* = .02, *BF* = 0.16; SP3, *d* = .32, *BF* = 1.53; *p* > .05 in all cases).

### Discussion

A number of findings resulted from Experiment 1. First, regarding performance by serial position, as expected, there was a general recency effect in which recall was superior for the most recently encoded item (item 3) relative to both the first and second item (e.g., Allen et al., 2017; Hitch et al., 2018, 2020). In addition, recall of the middle item was reliably poorer than the first, showing a particular vulnerability for that item, which was evident in both age groups. Under similar task conditions, but using recognition rather than recall to measure performance, Brown et al. (2017, Exp. 2) showed a marked dip in performance for older adults only. Despite the main effect of age in the present experiment, we suggest that the present requirement for recall appears to have elicited the same serial position effect in young adults, showing that the same vulnerability can be observed in young adults when using more challenging and sensitive performance measures.

Regarding ageing and prioritisation, an important finding was that both age groups were able to prioritise information in visual working memory. In this case, the prioritisation effect was clearest at serial position 2 where, based on the control condition, performance was most vulnerable. In both age groups, directing attention towards position 2 at encoding benefitted performance there over all others, specifically when position 2 was tested. Notably, though, and as expected (Atkinson, Berry, et al., 2018; Hu et al., 2014), when prioritising position 2, this resulted in a cost for performance at position 1, indicating how participants respond to the differential value of items by reallocating limited attentional resources towards high value and away from low-value items. Interestingly, no prioritisation benefit was observed for position 1, which somewhat contrasts with previous findings (Atkinson, Berry, et al., 2018; Hitch et al., 2018; Hu et al., 2014). However, it may be that participants’ approach to the task, in the absence of prioritisation instructions, was typically to direct their attention to boost position 1. This may partly explain why such a pronounced V-shaped pattern is observable on no-priority trials. Turning to the final sequence item, only a small and non-significant numerical prioritisation benefit was observed. While previous studies have indicated the presence of such an effect in young adults, this was reduced in magnitude compared to earlier serial positions (Hu et al., 2016). Thus, while participants may be able to prioritise the most recent item to a certain extent, the automatic boost that is typically experienced for this item (Allen et al., 2014) may mean that they are less able or less inclined to use attention to further increase its recall probability.

Finally, while older adults were able to boost their own performance by directing their attention towards certain items, the age effect was never reduced. This same outcome has been found previously in the context of availability of a semantic strategy in a visual matrix task, although in that case participants were not specifically instructed regarding strategy use (Nicholls & English, 2020; see also Hamilton et al., 2018, who reported no benefit of semantics in older adults). It is also in line with effects of value-directed remembering demonstrated by younger and older adults in the context of episodic long-term memory (e.g., Ariel et al., 2015; Siegel & Castel, 2018). This pattern of findings suggests that the associative deficit typically observed with ageing (Naveh-Benjamin, 2000; Old & Naveh-Benjamin, 2008) does not appear to be reduced with the value-based strategies that have been investigated thus far. Therefore, while the current benefit was indeed observed for older adults, this was of the same magnitude as for young adults, at least under these task conditions. This also fits with research showing that older adults experience similar sized visual cueing effects to younger adults (Gilchrist et al., 2016; Loaiza & Souza, 2018; Mok et al., 2016; Souza, 2016; Strunk et al., 2019). We next aimed to establish, given older adults’ generally lower performance levels, whether they could differentially benefit from the prioritisation strategy when allowing increased encoding time.

## Experiment 2

In line with Atkinson, Berry, et al. (2018), and Atkinson et al. (2019), each object in Experiment 1 was presented for 500 ms. While this is slightly longer than some other studies examining value-based prioritisation (e.g., 250 ms per item in Hitch et al., 2018; Hu et al., 2014, 2016), no study to date has directly manipulated encoding time per item in this context. It is possible that, with more time available during encoding, participants are better able to direct their attention towards the high-value item and produce larger performance benefits as a result. This might be particularly the case for older adults, as slowed processing speed has been shown to account for age effects in cognition (e.g., Salthouse, 1996; Verhaeghen & Salthouse, 1997), and working memory specifically (Brown et al., 2012; Salthouse, 1991; Vaughan & Hartman, 2010).

Experiment 2 therefore had two primary aims. First, we examined whether the observation from Experiment 1, of equivalent prioritisation benefits in younger and older groups, would replicate. Rather than examining each serial position, Experiment 2 focused on the control (no priority) and prioritise-SP2 conditions. This comparison yielded the most reliable priority effects in Experiment 1, and Brown et al. (2017) found a particular age-related deficit in remembering this middle object in a three-item sequence. Focusing on a single priority condition also reduces any possible confusion regarding which item to prioritise that might otherwise arise when the more valuable item changed in each block (as in Experiment 1).

Second, we examined the impact of varying encoding time between 500 ms (as in Experiment 1) or 1,000 ms per item on overall performance and on the magnitude of prioritisation benefits (Sander et al., 2011). In Experiment 1, encoding time may not have been sufficiently long for participants, and particularly for older adults, to be able to maximise gains from strategic prioritisation. Providing more time at encoding may help older adults compensate for possible reduction in processing speed. Therefore, we predicted that longer encoding times may enhance prioritisation benefits and that this would be particularly apparent for the older adult group.

### Method

#### Participants

There were 42 participants, none of whom had participated in Experiment 1. This included 24 younger adults (2 males, 22 females) aged 18–32 years (*M* = 19.88, *SD* = 2.72), from the University of Leeds student population, receiving course credit or payment for participation. There were 18 older adults (5 males, 13 females) aged 67–90 years (*M* = 74.33, *SD* = 6.70), who received payment for participation. The slightly lower number of older participants was due to a particular difficulty accessing participants in this age group at the time the study was conducted. Young adults achieved a mean NART IQ (Nelson & Willison, 1991) score of 113.04 (*SD* = 3.51), while older adults achieved a mean score of 120.89 (*SD* = 3.68). This was significantly different, *t* (40) = 7.04, *p* < .001, *d* = 2.2, *BF* > 10,000, and indicates the commonly observed advantage for older over younger adults in verbal knowledge. All older adults were classified as cognitively normal using the Mini-Cog assessment (Borson et al., 2000).

The University of Leeds School of Psychology granted ethical approval for this study (reference number: PSC-455).

#### Materials

All materials were the same as in Experiment 1.

#### Design and procedure

This experiment implemented a 2 × 2 × 2 × 3 mixed design, with age group as the between-subject factor, and prioritisation (control vs prioritise SP2), presentation time (500 ms vs 1,000 ms per object), and probed serial position (SP1, 2, or 3) as within-subject factors. The dependent variable was mean proportion correct in the cued recall task. Prioritisation and presentation time were manipulated in separate, counterbalanced blocks of 6 practice trials and 30 trials, with probed serial position implemented in random order within each block. Each SP was tested 10 times within each of the prioritisation × presentation time conditions (divided evenly between shape and colour probes). In total, there were 24 practice and 120 test trials in the experiment. Trial feedback was never provided, either within the practice or the experimental trials.

The visual working memory task was created in PsychoPy (Peirce, 2007) and presented on a 13-inch MacBook Air optimising full brightness, adjusted to eye level and placed approximately 50 cm away from the individual.

The trial procedure, including use of articulatory suppression, was similar to that implemented in Experiment 1, with two exceptions. First, presentation time varied between different blocks of trials, with each item presented for either 500 or 1,000 ms. Second, an inter-stimulus interval of 250 ms was included, mapping onto earlier work in this area (e.g., Atkinson, Berry, et al., 2018; Atkinson et al., 2019; Hitch et al., 2018; Hu et al., 2014, 2016). All participants gave written, informed consent prior to participation.

### Results

**Figure 5.**
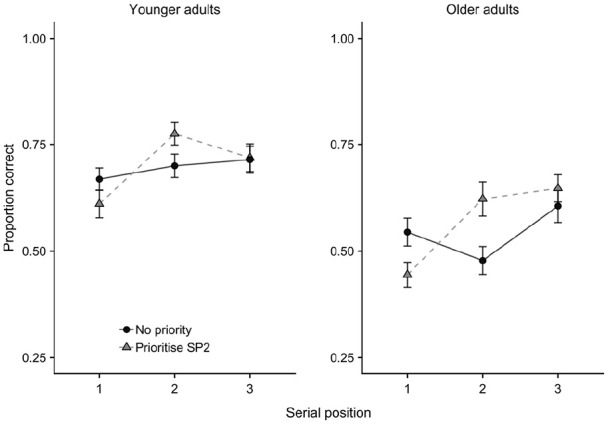
Mean proportion correct (with *SE*) for each age group, as a function of priority condition and serial position, in Experiment 2.

**Figure 6.**
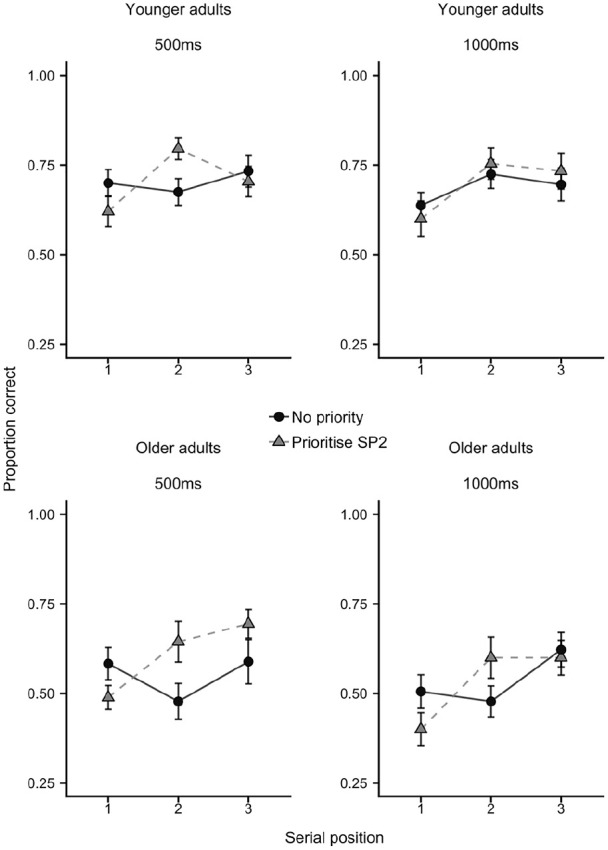
Mean proportion correct (with *SE*) for each age group, as a function of priority condition, presentation time, and serial position, in Experiment 2.

As in Experiment 1, performance was averaged across shape and colour probes to obtain a single cued recall measure. Mean proportion correct is displayed in Figure 5, collapsing across presentation time, and Figure 6, separated by presentation time.

A 2 × 2 × 2 × 3 mixed ANOVA indicated a significant effect of age group, *F*(1,40) = 15.42, *MSE* = 0.16, *p* < .001, $\eta_{p}^{2}=.28$, *BF* = 62.07, with younger adults (*M* = 0.71, *SE* = 0.03) more accurate than older adults (*M* = 0.57, *SE* = 0.03) overall. There was a significant effect of serial position, *F*(2,80) = 11.49, *MSE* = 0.04, *p* < .001, $\eta_{p}^{2}=.22$, *BF* > 10,000, which did not interact with age group, *F*(2,80) = 2.33, *MSE* = 0.04, *p* = .104, $\eta_{p}^{2}=.06$, *BF* = 0.68. There was no main effect of priority condition, *F*(2,80) = 1.0, *MSE* = 0.04, *p* = .32, $\eta_{p}^{2}=.02$, *BF* = 0.16, but we did observe the predicted interaction with serial position, *F*(2,80) = 12.35, *MSE* = 0.24, *p* < .001, $\eta_{p}^{2}=.24$, *BF* = 699, and indeed this was the only significant, BF-supported interaction to emerge across this analysis. Age group did not interact either with priority condition (*p* = .55, $\eta_{p}^{2}=.01$, *BF* = 0.18), nor was the age group × priority × SP interaction significant (*p* = .33, $\eta_{p}^{2}=.03$, *BF* = 0.18). There was no significant effect of presentation time, *F*(2,80) = 3.43, *MSE* = 0.03, *p* = .071, $\eta_{p}^{2}=.08$, *BF* = 0.41. Indeed, accuracy was slightly (but not meaningfully) lower in the 1,000 ms presentation trials overall, relative to the 500 ms trials (.62 vs .65). Furthermore, there was no evidence that time interacted with any other factor (*p* > .25, $\eta_{p}^{2}<.035$), with Bayes factors also supporting the null in each case (*BF* < 0.25). The one exception to this was the four-way interaction, with *BF* = 2.28, though this still represents uninformative evidence either way, especially when coupled with the non-significant frequentist outcome, *F*(2,80) = 1.42, *MSE* = 0.03, *p* = .25, $\eta_{p}^{2}=.03$.

To break down the significant priority × SP interaction, a set of (Bonferroni-Holm corrected) follow-up tests were carried out comparing control versus prioritise SP2 conditions at each serial position. This revealed the predicted advantage for the prioritisation condition (*M* = 0.71, *SE* = 0.03) over no priority (*M* = 0.61, *SE* = 0.03) at SP2, *t* = 3.18, *p* < .01, *d* = .49, *BF* = 12.08. Alongside this, there was a cost of prioritisation at SP1 (no priority *M* = 0.61, *SE* = 0.02; prioritise SP2 = 0.54, 0.03), *t* = 3.11, *p* < .01, *d* = .48, *BF* = 10.11, but no difference at SP3, *t* = 0.79, *p* = .43, *d* = .12, *BF* = 0.22 (no priority *M* = 0.67, *SE* = 0.02; prioritise SP2 *M* = 0.69, *SE* = 0.03).

### Discussion

The main findings from Experiment 1 were replicated, with older adults less accurate overall, relative to the younger group, but just as effective at prioritising an object within a sequence. Indeed, young adults produced a priority effect size at SP2 of *d* = .43, while older adults’ effect size was *d* = .56. This recall advantage for the high-value item was again accompanied by performance costs for the first item in the sequence, while the final item remained relatively unaffected.

For both age groups, presentation time had no effect, suggesting provision of more encoding and processing time does not help, either overall or in terms of prioritising a key, higher value item, at least under these task conditions. Thus, reducing time pressure did not particularly help older adults in this task. While this does not generally reject the processing speed theory of ageing, it does imply that speed of encoding is not a major limiting factor in the current context. However, it is worth noting that increased presentation time per item also resulted in longer retention time for early sequence items. Our current focus on visual working memory for sequences of items means that this was inevitable. It might therefore be fruitful for future work to examine the possible interaction between prioritisation and encoding time using simultaneous multi-item arrays. Indeed, Guest et al. (2015) showed that processing speed in ageing, indexed by the impacts of presentation time, was particularly important for multiple object arrays in visual working memory, and not for single objects. This would imply that increased presentation time is more useful in the context of simultaneously encountered arrays where multiple items must be encoded together.

## General discussion

Recent research has illustrated that both young adults (Allen & Ueno, 2018; Atkinson, Berry, et al., 2018; Hitch et al., 2018; Hu et al., 2014, 2016) and children (Atkinson et al., 2019) can prioritise more valuable information in working memory. Over two experiments, the present findings clearly demonstrate that these priority effects extend into older adulthood. Indeed, the younger and older adult groups appeared to be equally effective at prioritising, showing recall benefits for the higher value item and costs for the accompanying lower value items that were equivalent in magnitude. Thus, it was not the case that the requirement to prioritise one item in a sequence meant that older adults completely abandoned the other items or removed them from working memory. For both age groups, prioritising other items had a larger detrimental effect on the first item in the sequence. Participants may normally put more resources into maintaining this first item, while performance on the final item is somewhat protected by a relatively automatic recency boost (e.g., Allen et al., 2014; Hu et al., 2016).

Evidence that older adults can direct their attention to particular items in working memory with observable impacts on performance is in line with a selection of studies using other forms of manipulation. This includes asking participants to focus on a self-selected subset of items (Atkinson, Baddeley, et al., 2018, or directing attention using visually presented cues indicating which item is more likely to be tested (Gilchrist et al., 2016; Loaiza & Souza, 2018; Mok et al., 2016; Strunk et al., 2019; Souza, 2016). It also fits with research in episodic long-term memory showing that value-directed remembering effects remain constant with healthy ageing (e.g., Castel et al., 2002; Siegel & Castel, 2018), despite the overall effect of ageing not being reduced (as per the associative deficit hypothesis, Naveh-Benjamin, 2000; Old & Naveh-Benjamin, 2008; see also Ariel et al., 2015; Siegel & Castel, 2018). Note, further work would be useful to establish whether or not an even older (old-old) group of participants can benefit from value-directed remembering (see Castel et al., 2011). The present work is novel in illustrating that older adults are able to integrate differential item value into their task set and use this to strategically prioritise higher value items in working memory, in the absence of any visual cues.

These findings add to the literature in demonstrating how this form of strategic approach can be applied across the lifespan. While Berry et al. (2018) initially found that children aged 7–10 years showed no evidence of the ability to prioritise, Atkinson et al. (2019) did observe performance benefits for higher value items in this age group when the task context was adjusted to increase the child-friendly motivational aspects of the manipulation. Although this study did not apply these motivational features, we now have evidence that the ability to strategically focus on one item from a larger set of to-be-remembered stimuli, and show enhanced recall as a result, is observable in childhood and persists into older adulthood. Such effects emerge in the context of reduced visual working memory capacity in these age groups more generally, relative to the peak that is typically observed in young adulthood (Brockmole & Logie, 2013).

It has been suggested that this ability to actively prioritise a particular item in working memory is dependent on the availability of modality-general executive attentional resources (e.g., Hitch et al., 2020). This is supported by the observation that young adults show reduced or abolished prioritisation boosts in visual working memory when performing a concurrent verbal task with an increased attentional component (Hu et al., 2016). Similarly, within the context of visual search, Störmer et al. (2014) found that the reward benefit in search performance shown by older adults was not as large or consistent as that seen in younger adults and that all such reward effects were abolished by a concurrent working memory task. Assuming a somewhat reduced executive control ability in older adults (Braver & West, 2008; Reuter-Lorenz & Lustig, 2016), the present findings might run counter to the notion that prioritisation is critically dependent on this resource. If this were the case, older adults might have been expected to show a reduced benefit of prioritisation, analogous to patterns produced by younger adults under divided attention. Yet, across both experiments, older people were able to direct their attentional resources towards the intended object and boost performance levels for those objects. Indeed, older people have been shown to be able to compensate for (Cabeza et al., 2002), or “scaffold” (Reuter-Lorenz & Park, 2014), limited specialised neural resources (e.g., visual cortex) by incorporating more generalised processing resources (i.e., frontal cortex). Decreased neural specificity of functioning (or, “dedifferentiation”) predicts 30% of the variance in higher order cognition (e.g., J. Park et al., 2010). Furthermore, differential neural correlates of performance have been observed for older versus younger adults, with older adults’ task-specific resources being depleted sooner (i.e., at lower levels of task demand) than in young adults, requiring them to incorporate more generalised resources sooner (Carp et al., 2010). In this respect, the older brain may have to work more actively to achieve the same level of performance than a younger adult.

Ozimič and Repovš (2020) have recently proposed that ageing impacts on formation of working memory representations and on their active maintenance over time. Under this approach, performance on visual working memory tasks reflects an interaction between such components (see also Logie, 2011). Turning to the present study, on one hand, the observation that older adults are generally impaired on this visual working memory binding task, but not at boosting performance via active prioritisation, might only offer support for the former and not the latter component highlighted by Ozimič and Repovš (2020). However, active maintenance is likely an important component of the task in both the no priority and priority conditions. If anything, encouraging participants to strategically prioritise one of the memoranda might simplify the process of active maintenance by providing direction regarding how it should be applied, particularly as older adults do not appear to use as efficient strategies in visual working memory as do young people (e.g., Nicholls & English, 2020).

What might be the practical application of the present form of manipulation? Provision of guidance in how best to marshal limited attentional resources could offer a way of ameliorating age-related decline in working memory, by targeting these resources towards key to-be-remembered information. In this study, older adults were still less accurate overall compared to the younger group, in all experimental conditions (including for the higher value items). Thus, prioritisation did not remove or reduce the age-related deficit (Siegel & Castel, 2018). However, encouraging older adults to prioritise the item at the second serial position (where the manipulation had the largest effect, and where performance was otherwise least accurate) did remove the age deficit, when compared against the younger adults’ recall accuracy at this position in the no priority condition (Exp 1: *p* = .28, *d* = .32, *BF* = 0.47; Exp 2: *p* = .17, *d* = .43, *BF* = 0.65). While we would acknowledge that this is not a comparison of like for like, it does show that if older adults are directed to engage in strategic prioritisation for an item, this can remove the age-related memory deficit for that item. It is possible that this boost could be all the more beneficial for older people with cognitive impairments, with potential to boost performance above impairment levels. Future research should therefore explore whether strategic attentional direction is effective for different age groups and potentially with clinical groups, and using more ecologically valid, real-world task contexts.

## Notes

1. A 2 (age group) × 4 (prioritisation condition) mixed analysis of variance (ANOVA) showed no significant effects in articulation rate (all *p* > .36). The grand mean for articulations per trial was 5.52.
